# The impact of variable illumination on vegetation indices and evaluation of illumination correction methods on chlorophyll content estimation using UAV imagery

**DOI:** 10.1186/s13007-023-01028-8

**Published:** 2023-05-27

**Authors:** Yuxiang Wang, Zengling Yang, Gert Kootstra, Haris Ahmad Khan

**Affiliations:** 1 College of Engineering, China Agricultural University, Beijing, China; 2 Farm Technology Group, Wageningen University and Research, Wageningen, The Netherlands

**Keywords:** UAV images, Illumination correction, ELM, Automated multi-scale Retinex, Leaf chlorophyll content estimation

## Abstract

**Background:**

The advancements in unmanned aerial vehicle (UAV) technology have recently emerged as an effective, cost-efficient, and versatile solution for monitoring crop growth with high spatial and temporal precision. This monitoring is usually achieved through the computation of vegetation indices (VIs) from agricultural lands. The VIs are based on the incoming radiance to the camera, which is affected when there is a change in the scene illumination. Such a change will cause a change in the VIs and subsequent measures, e.g., the VI-based chlorophyll-content estimation. In an ideal situation, the results from VIs should be free from the impact of scene illumination and should reflect the true state of the crop’s condition. In this paper, we evaluate the performance of various VIs computed on images taken under sunny, overcast and partially cloudy days. To improve the invariance to the scene illumination, we furthermore evaluated the use of the empirical line method (ELM), which calibrates the drone images using reference panels, and the multi-scale Retinex algorithm, which performs an online calibration based on color constancy. For the assessment, we used the VIs to predict leaf chlorophyll content, which we then compared to field measurements.

**Results:**

The results show that the ELM worked well when the imaging conditions during the flight were stable but its performance degraded under variable illumination on a partially cloudy day. For leaf chlorophyll content estimation, The $r^2$ of the multivariant linear model built by VIs were 0.6 and 0.56 for sunny and overcast illumination conditions, respectively. The performance of the ELM-corrected model maintained stability and increased repeatability compared to non-corrected data. The Retinex algorithm effectively dealt with the variable illumination, outperforming the other methods in the estimation of chlorophyll content. The $r^2$ of the multivariable linear model based on illumination-corrected consistent VIs was 0.61 under the variable illumination condition.

**Conclusions:**

Our work indicated the significance of illumination correction in improving the performance of VIs and VI-based estimation of chlorophyll content, particularly in the presence of fluctuating illumination conditions.

## Introduction

Rapid population growth increases global food demand, which could be one of the greatest challenges in the coming decades. Accurate and timely crop monitoring is critical for farmers to make in-time decisions and optimize field management, increasing yield and thereby ensuring food security [1]. Recent advances in high-resolution imaging and unmanned aerial vehicle technologies enable high-throughput high-resolution monitoring of crop parameters [2]. Color images containing abundant spatial and color information [3] are widely used in UAV monitoring tasks. Vegetation indices (VIs) derived from RGB color images are treated as important features to get information about the status of the plants, for instance, to estimate the leaf chlorophyll content of maize and wheat [4] and to estimate the above-ground biomass of soybean [5].

While crop monitoring with the UAV is rapidly expanding, one inevitable issue is the variable illumination caused by different solar irradiation and fluctuating levels of cloud cover. In certain high-latitude regions, such as the Netherlands, the weather changes rapidly, and partially cloudy days are common. Additionally, longer flight campaigns increase the probability of encountering changes in illumination. As the incoming spectral irradiance to the camera mounted on the UAV is a combination of the characteristics of the plant and the solar spectral irradiance, UAV imagery obtained under variable illumination may provide misleading information about the crop. Observed differences in the image may be caused by actual crop variability or by changing lighting conditions. While some VIs are invariant to brightness, this is not sufficient to deal with the variable solar illumination, as cloud cover does not only change the brightness, but also the spectral properties of the illumination. Moreover, the solar spectral irradiance changes with the altitude angle of the sun, causing differences over the day. Therefore, UAV field investigations must consider illumination and compensate for it.

Existing approaches to compensate for the change in illumination for UAV color images can be classified as hardware or software methods [6–10]. Hardware methods require an additional sensor or reference targets. Mounting onboard upward-looking solar radiation sensors [11] is a common method for measuring solar irradiance and compensating for variations in lighting conditions. However, the UAV vibrations can affect the angle at which the sensor captures incoming light, leading to inaccurate measurement [12]. Moreover, mounting additional sensors increases the weight, costs, and complexity of the system. The empirical line method (ELM) is another frequently used approach in the UAV remote sensing field, in which multiple reference panels with known reflectance are placed on the ground to build a relationship between the RGB values of the image and the true reflectance [13, 14]. In this way, the illumination condition can be calibrated. However, this method assumes stable illumination throughout the flight, or always needs to have the reference panels in the camera view. As the latter is very impractical, it means that the method cannot be used under changing illumination conditions, for instance, during a partially cloudy day.

Besides the aforementioned hardware methods, several image-based methods have been developed for illumination correction of UAV images [15, 16]. Honkavaara et al. [15] proposed a radiometric block-adjustment method in which build correction equations using the difference for the same tie points in different consecutive images. However, it has been noted that the technique is susceptible to geometric distortions, the presence of pixels with radiometric anomalies, and the accumulation of errors [17]. Wang et al. [9] developed a tensor-decomposition technique to eliminate cloud shadows in UAV remote sensing images, considering their dynamic change over time. However, the method requires that illumination variability can be captured within images, which does not always hold. Additionally, invariant color models such as Retinex have been proposed to estimate the illuminant component in an image and to mitigate the impact of variable illumination on color images accordingly [18]. A multi-scale Retinex method has been devised to address the issues of color distortion and low contrast in underwater images [19]. Retinex combined with gamma correction enhances non-uniform illumination in close-range images [20] due to its constant color consideration. The advantage of Retinex over hardware-based illumination adjustment methods is that the change in illumination can be estimated based on the images themselves, which is convenient and cheap for UAV images. It furthermore does not assume that the drone itself is experiencing the same illumination as the plants on the field. To the best of our knowledge, Retinex correction has not been applied and tested in UAV applications and the effectiveness for agricultural applications is unknown.

Despite the aforementioned illumination-correction methods have been gradually developed for UAV imagery to ensure color consistency and accuracy under variable illumination conditions, there remains a knowledge gap regarding the influence of changeable illumination on different VIs, the performance of these methods in mitigating the impact of variable illumination, and their applications in agriculture, such as leaf chlorophyll content estimation. The presented study aims to improve this understanding. The paper has three objectives: (1) to assess the potential impact of variable illumination on sixteen RGB-VIs which are commonly used in UAV applications, (2) to investigate the performance of the ELM correction for UAV images collected under different illumination conditions and the usage of the corrected images to estimate the chlorophyll content of plant leaves, and (3) to evaluate the performance of Multi-scale Retinex correction under variable illumination conditions and the usage of the corrected images to estimate the chlorophyll content.

In the following section, the flowchart of data acquisition, processing, the selection of VIs, and the methods used to analyze the results are presented in detail. Afterward, in the section Result and Discussion, the experiments show the impact of illumination on VIs and the performance of illumination correction methods. Based on these results we provide some guidelines for illumination correction in UAV RGB images.

## Method

### Field experiments

**Fig. 1:**
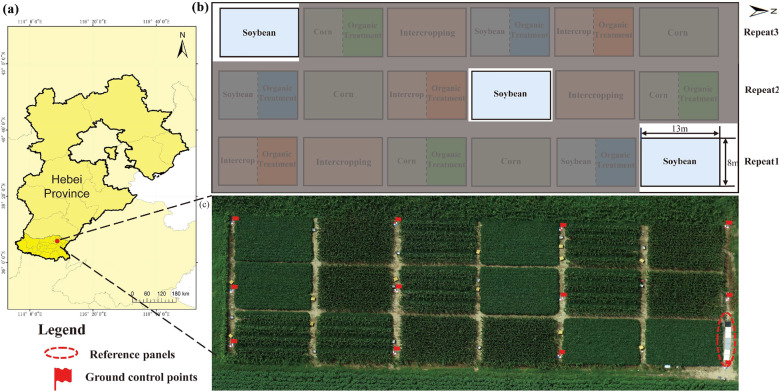
**a** Location of the study area in Hebei, China **b** An overview of the study site and three soybean plots and **c** an RGB map of the experimental field, and the setup of 12 GCPs and reference panels

The field experiment was carried out in August, 2021 at the Quzhou Experiment Station of China Agricultural University situated in Quzhou County, Hebei Province, China $(36{^\circ } 51{^\prime } 45{^\circ } {N},115{^\circ } 00{^\prime } 57{^\circ } {E})$ (Fig. 1a). Different planting systems were tested on the experimental site, and the experiment was set up with three replicates, yielding a total of 18 plots. In this study, the monoculture field of soybeans (Fig. 1b) was selected for analysis due to the uniform growth conditions of the soybean plants. Data was collected at the mature stage of soybean growth, when the plants had formed a dense canopy.

To guarantee precise geolocation of the UAV images on various dates, 12 ground control points (GCPs) were strategically placed in the field (Fig. 1c). The center point coordinates of 12 GCPs were measured with a geodetic-dual frequency global navigation satellite system (GNSS) receiver with a precision of 0.02 ms in both the horizontal and vertical dimensions. Furthermore, two ethylene-vinyl acetate (EVA) greyscale reference panels with a reflectance of 3.5%, two EVA panels with a reflectance of 20%, two panels with a reflectance of 80% and one panel with a reflectance of 84.5% were placed at the north corner of the field (Fig. 1c). These panels are used for the calibration of the images. The calibration method is discussed in Section Empirical Line Correction .

### Data acquisition

#### Flight campaigns

In the study, aerial images were obtained using a Professional Phantom 4 RTK quadcopter from DJI (Shenzhen, China). This UAV was equipped with a 1-inch FC6310 CMOS sensor and an aerial-optimized f/2.8 wide-angle lens with a 24 mm equivalent focal length and produces images of $5472\times 3648$ pixels [21]. Furthermore, the RTK receiver installed on the quadcopter ensured flight stability and provided centimeter-level mapping accuracy on image metadata during flights at an altitude of 100 m. This allowed for a ground spatial resolution (GSD) of 2.7 cm to be achieved.

UAV images were obtained during varying illumination conditions, including sunny skies, complete cloud coverage, and partly cloudy skies, between August 14th and 18th, 2021. Each flight was conducted between 11:30 and 13:00 when the solar elevation angle was almost at the peak. The camera was in manual mode, and setting parameters were adjusted manually to capture the illumination change and avoid saturated pixels. For each flight, the camera was configured with a normalized white balance, an aperture of F5.6, a shutter speed of 1/500, and an ISO value of 100. One hundred five images covering the entire experimental site were acquired at 25 m above ground level (0.68 cm GSD) for each flight mission with a 1.2 m/s flight speed. Details on these flights are shown in Table 1. Meanwhile, the flight route was planned with 80% heading overlaps and 70% side overlaps to achieve high-quality image mosaics [22].

**Table 1:** Information on the flight campaigns: date of image collection, the illumination condition, and the time-span of image acquisition

Date	Illumination condition	Collection time
14-August-2021	No cloud (sunny)	12:01–12:10
15-August-2021	Partly-cloudy (variable)	11:48–11:56
16-August-2021	Cloudy	12:15–12:23

#### Ground measurement

On the 14th of August, leaf chlorophyll content was measured on the same day after the UAV campaigns. Four plants were selected randomly from each of the three plots, and their geographic locations were recorded. The chlorophyll content was determined using a Soil Plant Analysis Development (SPAD) 502Plus Chlorophyll Meter (Konica-Minolta, Japan). The SPAD value, which is a strong indicator of chlorophyll content, was obtained through the measurement of leaf absorbance at two wavelengths (650 and 940 nm) [23, 24]. The SPAD readings for each soybean plant were determined by averaging the measurements obtained at three different positions on each of the top five leaves, yielding a single, representative SPAD value for each plant. No significant differences were observed in the chlorophyll content of soybean plants within the same plot. A low degree of variability is displayed in Table 2, indicated by the coefficient of variation (CV) and standard deviation (Std).

**Table 2:** Statistical summary of the in-situ measured leaf chlorophyll content of soybeans in monoculture plots using a SPAD-502Plus

Date	Min	Mean	Max	Std	CV%
14-August-2021	40.9	47.25	54.1	3.00	6.35

### Data processing

#### Flowchart of data processing

**Fig. 2:**
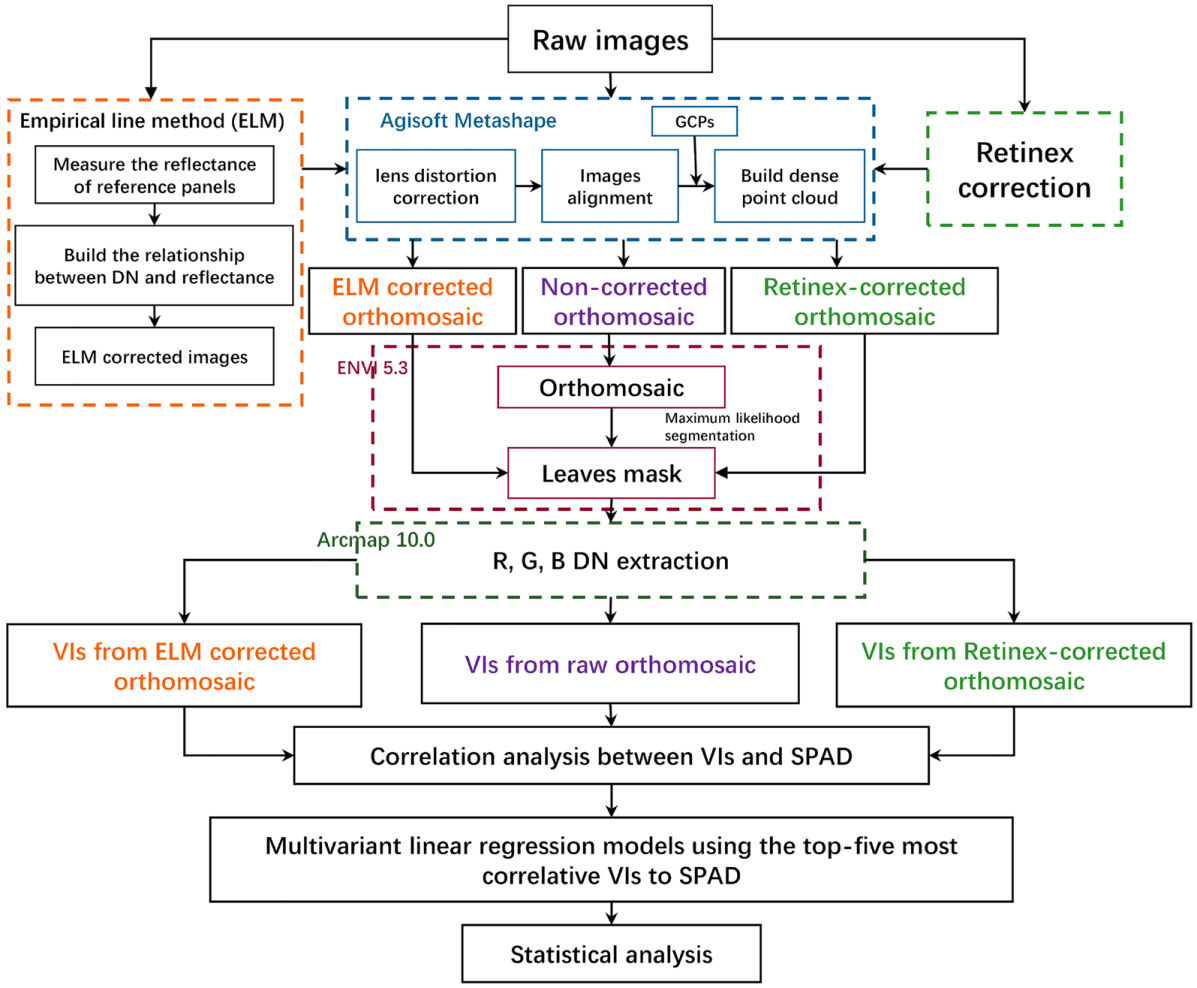
Flowchart for UAV image processing and analysis including illumination correction (ELM and Retinex correction), orthomosaic creation, leaves segmentation, VIs extraction, and linear regression modeling from VIs to SPAD. The workflow is o process aerial images collected under sunny, overcast and cloudy conditions with variable solar irradiance. The ELM correction was only performed in sunny and overcast conditions

The data processing pipeline is illustrated in Fig. 2. Firstly, the illumination correction of single raw aerial images was conducted by ELM and Retinex correction, separately. These methods are elaborated in sub-sections Empirical Line Method  and Automated Multi-scale Retinex correction,  respectively. Then, the corrected images, taking into account changes in illumination, were brought into Agisoft Metashape software (version 1.5.1, created by Agisoft LLC in St. Petersburg, Russia) for geometric correction using the Brown-Conrady method and pre-calibrated parameters. The software employed a structure-from-motion (SFM) method to stitch images together by estimating the position and orientation of the camera through the process of bundle adjustment, which is based on looking for matching features in overlapping images [25]. The process of aerial triangulation, dense point cloud construction, mesh building, digital surface model, and orthomosaics generation in Agisoft is shown in Fig. 2.

After generating orthomosaics, the orthomosaic were processed using Maximum likelihood classification (MLC) in Exelis Visual Information Solutions (ENVI, 5.3) to identify the vegetation areas on the UAV orthomosaic of all flights. Then, a square buffer with a length of 0.2 m for each sampling crop was generated in ArcMap (Environment System Research Institute, ESRI, 10.0) and the leaf area of each sampling crop was derived by intersecting those buffers with leaves masks. Afterward, the zonal statistics plugin in ArcMap was utilized to obtain the average values of each VIs within the leaf regions of each crop sample. The VIs are introduced in section. A total of 12 soybean plants were selected to extract VIs. Lastly, multivariate linear regression models using the top-five most relevant VIs were built to relate the VIs with the in-site SPAD values, and statistical analysis was performed as described in Section Evaluation. 

#### RGB-based vegetation indices

Vegetation indices, which are important features for remote sensing, are frequently used in satellite images for detecting changes in crops [26]. Besides, with the development of UAV technology, a range of RGB-based vegetation indices have also been developed for UAV images. [4, 27, 28]. According to the way of calculation, VIs are divided into ratio VIs and difference VIs. In this study, six difference VIs and ten ratio VIs are investigated as shown in Table  3. The excess green index (EXG) was commonly used for automatically green crop extraction [29] and assessing the vigor of green vegetation [30]. To highlight and better take advantage of the greenness component which is considered as an indicator of crop status, excess green minus excess red (EXGR) was developed by subtracting the excess red index (EXR) from the green component. Similarly, the color indices of vegetation extraction (CIVE) accentuate the green component of the images through the use of principal component analysis on the information contained in RGB bands. Additionally, the green-red ratio index (GRRI) was frequently employed to analyze the sensitivity of VIs to complex canopy structures. Normalized green-red difference index (NGRDI), normalized green-blue difference index (NGBDI), and modified green red vegetation index (MGRVI) were designed as a phenology indicator and have been used to estimate biomass [31] and assess drought tolerance [32]. Visible-band difference vegetation index (VDVI) was designed to extract green vegetation. Furthermore, vegetation index (VEG) was also used to identify green vegetation, and VEG was found to be less sensitive to complex illumination conditions [33]. Besides, the combination of the green index (COM) was used to analyze plant characteristics by combining three indices that focus on the greenness aspect. Visible atmospherically resistant Index (VARI) provided good performance in vegetation fraction estimation while mitigating the influence of atmospheric effects [34]. The last two VIs, the Kawashima index (IKAW) and the RGB Vegetation Index (RGBVI), have also been employed to observe the growth of wheat and predict wheat yield, as reported in Woebbecke’s study [35]. The potential influence of illumination on each VI was assessed. Meanwhile, the impact of illumination on the relationship between VIs and chlorophyll content was investigated using the Pearson correlation coefficient. In addition, the impact of variable illumination on VI-based leaf chlorophyll content estimation was also assessed and presented in the Results section.

In this study, r, g, and b values were normalized to the range from 0 to 1 based on the following two ways separately denoted as scaling ($r_s$, $g_s$, $b_s$) and normalization ($r_n$, $g_n$, $b_n$). The calculation method of scaling represents raw data without any processing, while normalization represents a way of band normalization.

Scaling:

1 $$\begin{aligned} \small r_s= \dfrac{R}{R_{max}}, b_s= \dfrac{B}{B_{max}}, g_s= \dfrac{G}{G_{max}}, \end{aligned}$$

Where $R_{max}= B_{max}=G_{max}=255$ for 8-bit color images.

Normalization:

2 $$\begin{aligned} \small r_n= \dfrac{R}{R+G+B}, b_n= \dfrac{B}{R+G+B}, g_n= \dfrac{G}{R+G+B}, \end{aligned}$$

**Table 3:** Overview of RGB-based vegetation indices derived from UAV images in this study (r, g, and b are related to the scaling and normalized values)

Index	Name	Formulation	References
E1	Excess Green (EXG)	$2g-r-b$	[36]
E2	Excess Red (EXR)	$1.4r-g$	[37]
E3	Excess Blue (EXB)	$1.4b-g$	[38]
E4	Excess green minus excess red (EXGR)	$EXG-EXR$	[39]
E5	Green blue difference (GBDI)	$g-b$	[40]
E6	Color index of vegetation extraction (CIVE)	$0.441r-0.811g+0.385b+18.78745$	[41]
E7	Green-red ratio index (GRRI)	*g*/*r*	[42]
E8	Normalized green red difference index (NGRDI)	$(g-r)/(g+r)$	[43]
E9	Normalized green blue difference index (NGBDI)	$(g-b)/(g+b)$	[44]
E10	Modified green red Vegetation Index (MGRVI)	$(g^2-r^2)/(g^2+r^2)$	[45]
E11	Visible-band difference vegetation index (VDVI)	$(2g-r-b)/(2g+r+b)$	[46]
E12	Vegetative index (VEG)	$g/r^\alpha b^(1-\alpha )$	[33]
E13	Combination of green (COM)	$0.36EXG+0.47CIVE+0.17VEG$	[47]
E14	Visible atmospherically resistant index (VARI)	$(g-r)/(g+r-b)$	[34]
E15	Kawashima index (IKAW)	$(r-b)/(r+b)$	[48]
E16	RGB Vegetation Index (RGBVI)	$(g^2-b\times r)/(b^2+b\times r)$	[45]

#### Empirical line correction (ELM)

The empirical line correction method is the most widely used method for UAV imagery. This is to convert the raw sensor data into relative reflectance and to correct for intensity in solar irradiance. In this study, the performance of ELM on mitigating the impact of illumination on VIs-based leaf chlorophyll content estimation was investigated.

**Fig. 3:**
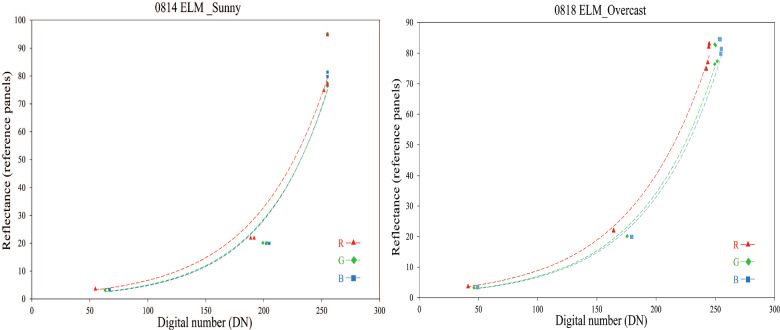
Empirical line method illumination correction curve based on reference panels. The left-hand figure is for a sunny day (14th, August) and the right-hand figure is for an overcast day (18th, August). The X-axis represents the image raw DNs of panels captured under each scenario, and the y-axis is the reflectance of reference panels

In this study, the ethylene-vinyl acetate (EVA) greyscale reference panels were chosen for calibration comprehensively considering their affordability and feasibility. In Jeong’s study [49], the feasibility of using EVA mats for UAV RGB imagery ELM correction was evaluated. Seven small gray-scale panels were utilized in the experiment to generate several spectral correction curves based on low-altitude UAV-RGB images captured before each flight on all dates except August 15th, due to the variable solar irradiance that day. The ELM method requires uniform illumination during the flight for proper correction, and abrupt changes in illumination can reduce its effectiveness [50]. Therefore, the ELM correction was only applied to orthomosaics collected under fully sunny and overcast conditions to ensure optimal correction results. Figure 3 shows the correction curves that demonstrate an exponential relationship between digital numbers (DNs) and reflectance for each color channel [14]. The correction equations are exemplified in Table 4.

**Table 4:** Regression equations between DN values of panels and reflectance values

Date	Camera waveband	Regression equation	Goodness-of-fit ($R^2$)
14th August-Sunny	Red	$1.35\times e^{0.016x}$	0.959
	Green	$0.877\times e^{0.017x}$	0.943
	Blue	$0.817\times e^{0.018x}$	0.951
18th August-Overcast	Red	$1.947\times e^{0.015x}$	0.998
	Green	$1.403\times e^{0.016x}$	0.991
	Blue	$1.351\times e^{0.016x}$	0.993

#### Automated multi-scale retinex correction

The ELM method is not suitable for the scenes where there is a change in the illumination during the data acquisition because it typically takes place only once before or at the end of the flight, we investigated image-based methods to tackle this challenge. Inspired by the literature on color constancy, the Retinex Theory was chosen as a potential solution. It was proposed by Land and McCann [18]. After decades of development, Retinex correction has made a significant impact in adjusting uneven illuminated color images. The foundation of the Retinex theory is the concept that the observed image ($S_{(x,y)}$) is a combination of the reflectance component ($R_{(x,y)}$) and the illumination component ($L_{(x,y)}$), which can be expressed as follows in Eq. (3). Through the implementation of a logarithmic transformation, the reflectance of the object can be computed using Eq. (4), effectively separating the influence of illumination on the image from the reflection of the surface. The benefit of using Retinex correction on UAV images is that it does not require any additional equipment, as the illumination component is estimated from the images themselves, providing a cost-effective solution.

3 $$\begin{aligned}{} & {} S_{(x,y)} = L_{(x,y)} \times R_{(x,y)} \end{aligned}$$

4 $$\begin{aligned}{} & {} \lg (R_{(x,y)}) = \lg (S_{(x,y)})- \lg (L{(x,y)}) \end{aligned}$$

5 $$G_{(x,y)} = \dfrac{1}{2\pi \sigma ^2}exp\,\left(-\dfrac{x^2+y^2}{2\sigma ^2}\right)$$

The advancement of Retinex technology has progressed from Single-scale Retinex (SSR) to Multi-scale weighted average Retinex (MSR) and then to the Multi-scale Retinex with color restoration (MSRCR) algorithm. SSR, first proposed by Jobson [51], estimates the illuminance component by convolving the original image with a Gaussian low-pass filter, as represented by Eq. (5). The scale parameter ($\sigma$) in the Gaussian filter is used to balance the preservation of color and texture details in the image. The MSR algorithm modulates the value of $\sigma$ to achieve various illumination enhancement effects and prevent color distortion. In this study, the automated MSR algorithm developed by [52] was selected to correct the illumination component in a single UAV image. This algorithm adjusts $\sigma$ automatically and performs well in producing an illuminant-invariant representation of images captured under variable illumination conditions. After adjusting all the images, they were imported into Agisoft to generate the illumination-corrected orthomosaic. We analyzed the difference in the performance of normalization of images, scaling, ELM correction, and Retinex illumination correction on the estimation of chlorophyll content of leaves. The analysis methods are described in the following section.

### Calculation of VIs for specific plants using four methods

As described above, the impact of illumination on images was corrected by two correction methods (ELM, Retinex) and the vegetation indices were normalized in two ways. In summary, VIs were calculated using four different methods:

1. The raw images were normalized by scaling equations as described in Eq. (1) and the VIs were calculated on the orthomosiacs for the leaves of the target plants,
2. The raw images were normalized by the normalization method described in Eq. (2), and VIs were calculated on the orthomosiacs for the target plants,
3. The raw images were corrected by the ELM, then normalized using Eq. (2) and the VIs were calculated,
4. The raw images were corrected by the automated multi-scale Retinex algorithm, then normalized using Eq. (2) and the VIs were calculated.

### Evaluation

The methods are evaluated in three ways:

- To assess the impact of variable illumination on VIs, the standard deviation (std) over the twelve sample plants of each VIs under different illumination conditions was calculated. If the influence of different illumination is mitigated well, the standard deviations for a specific VI should be similar for the different illumination conditions. If this is not the case, the std’s should differ and specially the std for the variable-illumination condition is expected to be much larger.
- To investigate the correlation between each VI extracted from the plants and the associated manually measured SPAD value, the Pearson correlation coefficient was calculated according to Eq. (6). The correlation coefficient were then compared for the four different methods and on the three different illumination conditions.
- To evaluate how well the leaf chlorophyll content could be predicted from the VIs, multivariate linear regression models were built using the top-five most relevant (highest correlation) VIs [4, 28]. The number of VIs included in the models was restricted to five to prevent overfitting. The performance of the models was evaluated using the coefficient of determination, $R^2$ and compared for the different methods and illumination conditions.

6 $$\begin{aligned} P = \dfrac{\sum _{i=1}^{n=12}{(x-{{\bar{x}}})} \times {(y-\bar{y})}}{\sqrt{\sum _{i=1}^{n=12}{(x-{{\bar{x}}})}^2\sum _{i=1}^{n=12}{(y-\bar{y})}^2}} \end{aligned}$$

Where *x* represents each vegetation index (VI) and *y* represents the SPAD values. ${{\bar{x}}}$ and ${{\bar{y}}}$ are the average of the respective measurements. The linear regression models were then evaluated using the coefficient of determination ($R^2$) as following Eq. (7).

7 $$\begin{aligned} R^2= \dfrac{\sum _{i=1}^n{(y_{i}-{\bar{y}})} \times {({\hat{y}}_{i}-{{\bar{\hat{y}}}})}}{\sqrt{\sum _{i=1}^n{(y_{i}-{{\bar{y}}})}^2}\sqrt{\sum _{i=1}^n{({\hat{y}}_{i}-{\bar{\hat{y}}})}^2}} \end{aligned}$$

In this equation, *n* represents the total number of samples, $y_i$ represents the true values, and ${\hat{y}}_i$ represents the predicted values. ${\bar{y}}$ and $\bar{{\hat{y}}}$ are the mean of *y* and ${\hat{y}}$, respectively.

## Results

### The impact of illumination on VIs

**Fig. 4:**
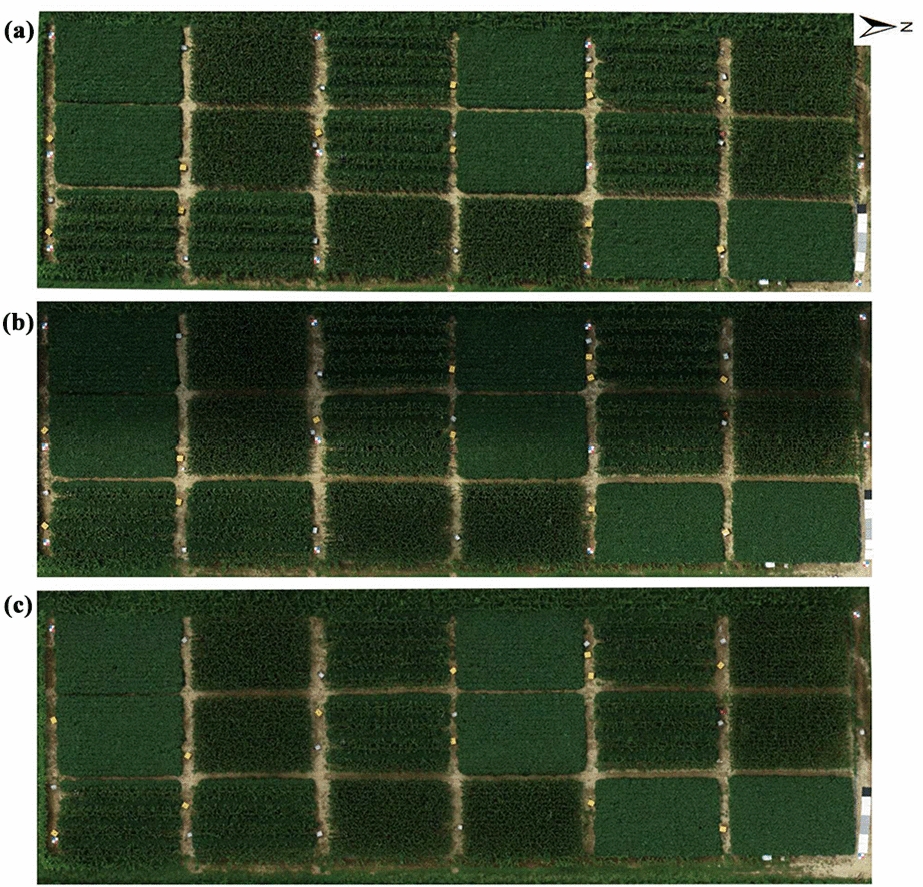
The RGB orthomosaics collected under three different illumination conditions: **a** the orthomosaic collected on the clear sunny sky with a stable solar irradiance, 14th August. **b** the orthomosaic collected on a semi-cloudy day with variable solar irradiance, 15th August, and **c** the orthomosaic collected under the totally overcast day, 18th August

Figure 4 presents three orthomosaics collected under different illumination conditions on sunny, variable solar irradiance and overcast days. Figure 4a and Fig. 4c illustrate the RGB data captured under a clear sunny day and an overcast day with consistent cloud coverage, respectively. These consistent lighting conditions provide better color consistency and radiometric accuracy. Under such ideal conditions, the estimation of illumination and therefore, correction for its influence is not needed. However, such ideal conditions are not present all the time and there might be times when data collection is needed despite having varying illumination conditions. Figure 4b shows the data captured on a partially cloudy day with sharp transitions from the sun to shadow during the flight. Such conditions result in inconsistent representation as some parts are acquired in sunny conditions and some parts are in the shadow of clouds. This is a frequent phenomenon for low-altitude UAV imagery. Such inconsistencies negatively affect the composites and vegetation indices extracted from the dataset, thereby causing errors in the leaf chlorophyll content estimation.

**Fig. 5:**
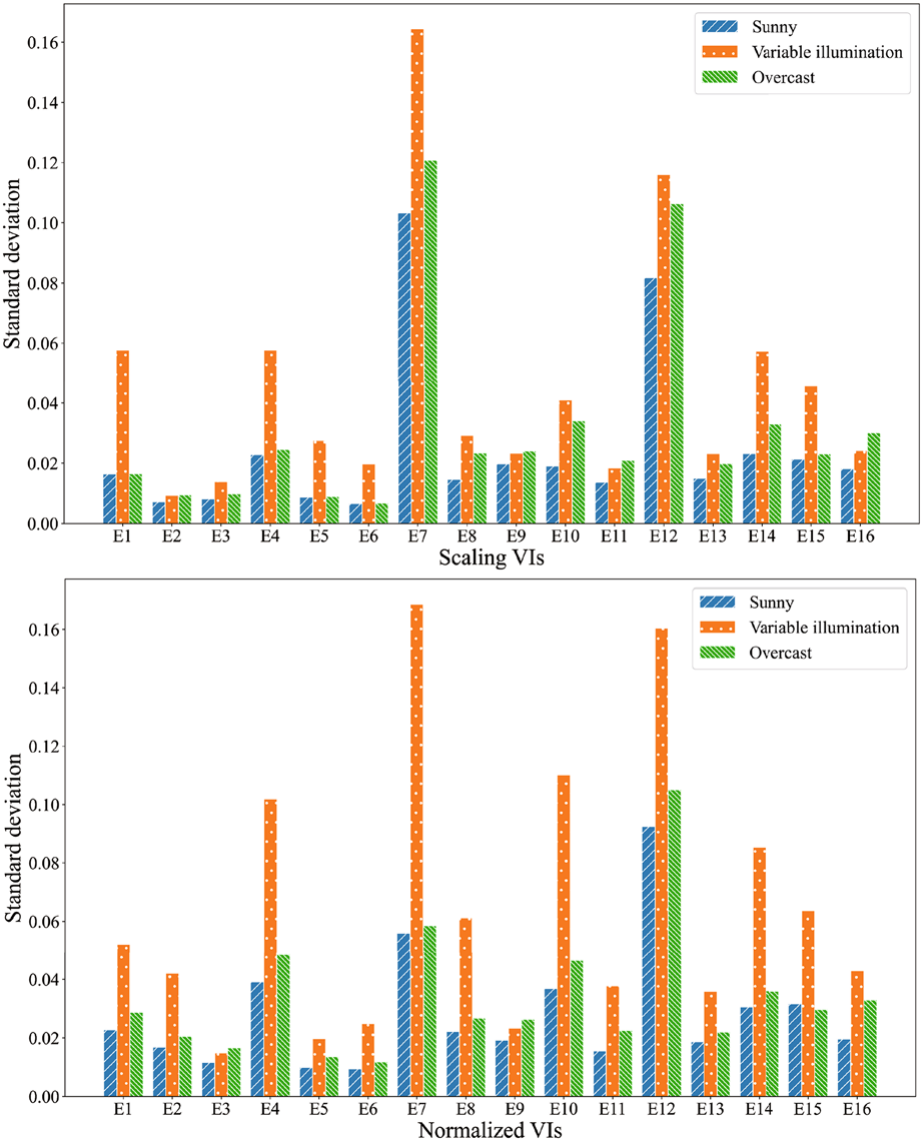
Standard deviation of all the vegetation indices for soybeans under three different illumination conditions (sunny, overcast, and variable solar irradiance conditions)

Figure 5 illustrates the standard variation of 16 chosen VIs under different illumination conditions. It can be observed that there is a noticeable increase in the standard deviation under variable illumination for all scaled and normalized VIs, in comparison to the consistent lighting conditions (sunny and overcast). This result highlights the significant impact of illumination on VIs. For example, the scaling Excess Green (E1) index showed a range of 0.0164 and 0.0165 under sunny and overcast conditions, respectively, while the range increased to 0.058 under variable illumination. This increase in dispersion of the E1 index indicates that the varying illumination causes unexpected anomalies in the data, negatively impacting the estimation of crop traits using VIs. On the other hand, several VIs, such as scaling E2, E9, E11, and E16, demonstrate more robust performance in resisting the influence of variable illumination. Similarly, normalized E3 and E9 exhibit a similar trend, with a limited increase in their range under variable solar irradiance. This result suggests that the robustness of VIs to variable illumination is index-dependent. Therefore, it is important to take the robustness of VIs into consideration when estimating crop traits.

To further understand the impact of illumination on the use of VIs, Fig. 9 presents the Pearson correlation trends between VIs and SPAD values under different illumination conditions including sunny, overcast, and variable illumination. For scaling VIs, the maximum value of Pearson correlation under the sunny condition was 0.57 (E1 and E6). The maximum value of Pearson correlation was 0.38 (E15) under the consistent low illumination condition. For E1 and E6, the Pearson correlation was 0.37, which is close to the maximum value. However, a significant decrease was observed under the variable solar irradiance condition and the maximum value of Pearson correlation decreased to 0.26 (E2). This suggests that variable illumination has a negative effect on the correlation between VIs and leaf chlorophyll content. The trend was similar for both normalized and scaling VIs, with the correlation being strongest under sunny conditions, and weakest under variable illumination conditions. Additionally, the performance of leaf chlorophyll content estimation using multivariant linear models built with the top-five most relevant VIs is shown in Table 5. It was observed that the $R^2$ of the model built based on scaling VIs under the sunny condition is 0.68. However, there was a significant decrease in $R^2$ under overcast and variable illumination conditions ($R^2$ is 0.33 and 0.30, respectively). The $R^2$ of the model built by normalized VIs indicated an unpredictable variation. The $R^2$ was 0.53 under the sunny condition, while it increased to 0.74 under the overcast condition. Nonetheless, the $R^2$ of the model decreased to 0.12 under the variable illumination condition. The result indicates that changes in illumination conditions significantly impacted the performance of the leaf chlorophyll content estimation models and that the performance was unpredictable and inconsistent if illumination correction was not applied to UAV images. These results emphasize the importance of illumination correction for accurate and reliable crop traits estimation from UAV imagery.

### The performance of ELM correction on VIs and chlorophyll-content estimation

The ELM correction is commonly used in UAV applications to convert DN into surface reflectance [8] and correct the intensity of solar irradiance across different consistent illumination conditions. The performance of the ELM correction on VIs and estimation of leaf chlorophyll content were investigated under different illumination conditions. As mentioned before, the ELM method is not able to deal with the variable illumination during the flight because the panel calibration usually happens before or after the flight [8]. Thus, the following analysis of the ELM method was conducted using data collected under sunny and overcast conditions only.

**Fig. 6:**
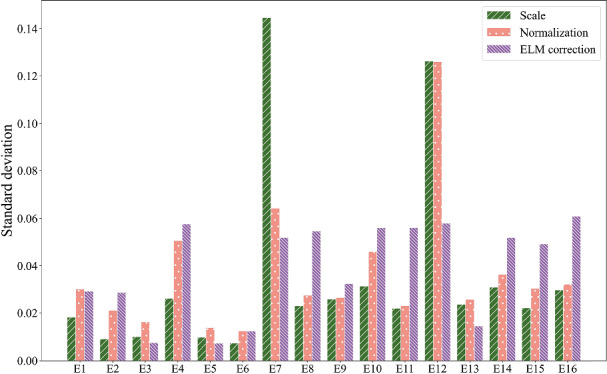
Standard deviation of scaling, normalized and ELM corrected VIs combining sunny and overcast datasets

To illustrate the impact of the ELM correction on VIs, the VIs collected under sunny and overcast conditions were combined together and obtained their standard deviation comprehensively. The standard deviation values are illustrated in Fig. 6. There was an increase in Std for half of all the VIs (E2, E4, E8, E9, E10, E11, E14, E15, and E16). However, it showed a significant decrease in Std for E7 and E12 when a significant difference appears in uncorrected VIs. Furthermore, to assess the performance of the ELM correction on leaf chlorophyll content estimation, the Pearson correlation of each ELM corrected VI and SPAD value is observed in Fig. 9. The Pearson correlation of each VI and SPAD significantly improved after ELM correction under the sunny condition. The average value of Pearson correlation was 0.28 for scaling VIs and 0.32 for normalized VIs under the sunny condition. However, the average value of correlation rose to 0.68 after ELM correction. Besides, the average value of Pearson correlation was 0.3 for scaling VIs and 0.22 for normalized VIs under the overcast condition. After ELM correction, the average value of correlation increased to 0.35, but not as much as it did under sunny conditions. The ELM correction helps improve the correlation between VIs and SPAD, especially under sunny conditions. Moreover, it can be observed from Table 5 that the performance of the multivariant model built by the top-five most relevant VIs became more stable after the ELM correction. The $R^2$ of the model was 0.60 under sunny conditions and 0.56 for overcast conditions. The result demonstrates that the ELM correction helps improve the repeatability of the leaf chlorophyll content estimation model across different dates.

In summary, the ELM correction improves the repeatability of the crop traits estimation model when the imaging conditions are uniform across the field. The result demonstrates the importance of factoring in the actual solar irradiance for the flight, especially when flights are conducted across dates under different illumination conditions.

### The result of multi-scale retinex correction

**Fig. 7:**
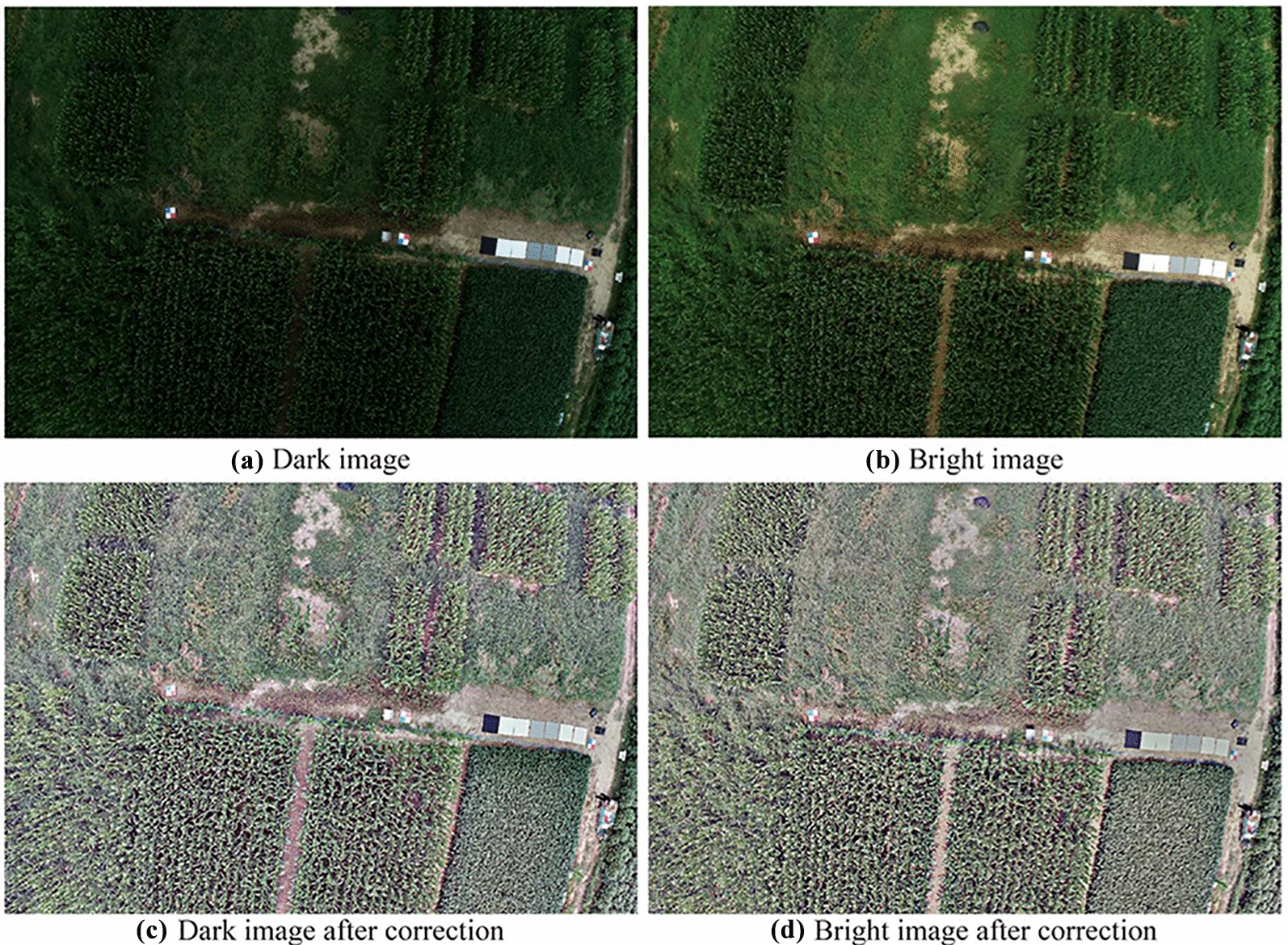
The result of automated multi-scale Retinex correction. **a** The image collected under clouds shadow, and **b** the bright image was collected consecutively after the dark image when solar irradiance suddenly increased, **c** the illumination corrected dark image, and **d** the bright image after the illumination correction

Aiming at the shortcomings of the ELM method, automated multi-scale Retinex correction was implemented on UAV images collected acquired during flights with varying solar irradiance. Figure 7 presents the result of Retinex correction. In the figure, two consecutive images taken on the same route, but with a significant change in solar irradiance from shadow to sunny, are shown in Fig. 7a and b. This change in illumination led to a noticeable difference in the color of the crop canopy, making crop traits estimation based on color unreliable. Figure 7c and d show the illumination-corrected images using the automated multi-scale Retinex method. The reason for applying Retinex correction on both images was that they were both affected by the change in illuminations. As can be seen from the figure, the brightness of both images was corrected to almost the same level and the difference between the two images decreased. This correction results in obtaining consistent RGB values across the field and thus consistent VIs can be computed. It also helps in making sure that a change in VI values indicates a change in the crop’s condition and is not caused due to the change in illumination. In conclusion, the use of automated multi-scale Retinex correction helps to mitigate the impact of illumination on images and achieve color constancy across images during one flight.

**Fig. 8:**
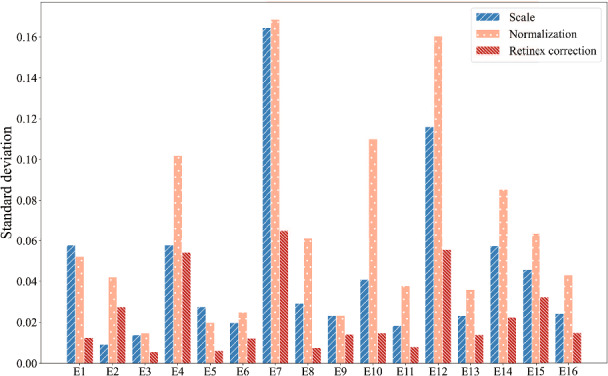
Standard deviation of all the scaling VIs, normalized VIs, and Retinex corrected VIs under the variable solar irradiance condition

By addressing the change of illumination, the consistency of VIs is also assessed. Figure 8 illustrates the standard deviation of scaling VIs, normalized VIs and automated multi-scale Retinex corrected VIs extracted from the orthomosaic captured under the variable solar irradiance condition. Compared with scaling VIs and normalized VIs which show an intense fluctuation together with a change of illumination, the Retinex correction achieves good performance on consistent representation of VIs. All automated multi-scale Retinex-corrected VIs exhibit a decrease in Std, indicating a smaller discrete degree of corrected data. This demonstrates the effectiveness of the Retinex correction in mitigating the influence of illumination on VIs and in achieving color consistency across images captured under variable solar irradiance conditions.

**Fig. 9:**
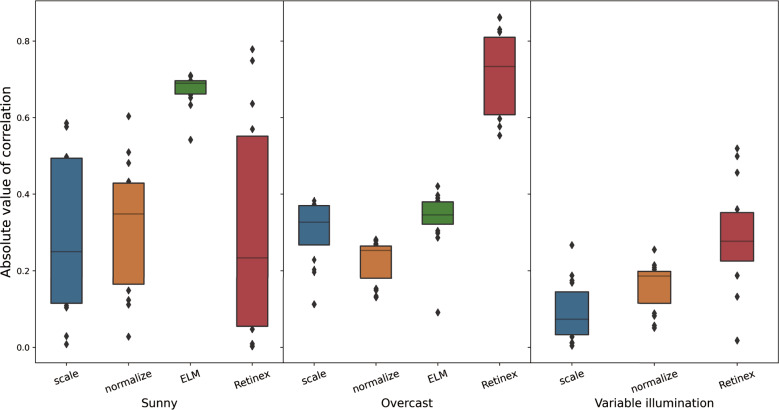
Boxplot of the absolute value of Pearson correlation of each VI (scaling VIs, normalized VIs, ELM corrected VIs and Retinex corrected VIs) and SPAD values. The VIs of soybean was extracted from the orthomosaic captured under three illumination conditions (sunny, overcast, and variable illumination condition)

To further assess the importance of consistent color on leaf chlorophyll content estimation, the Pearson correlation of corrected VIs and SPAD values was calculated. The results, as depicted in Fig. 9, show that the average Pearson correlation between scaling VIs, normalized VIs, and SPAD maintained at a low level under variable illumination conditions. The average absolute value of correlation for scaling VIs and normalized VIs were 0.09 and 0.16, respectively. The result indicates the negative impact of varying illumination during the data acquisition on VIs, and thereby the crop leaf chlorophyll content estimation in the following stage. However, after applying Retinex correction, the correlation between corrected VIs and SPAD values increased significantly, with an average correlation of 0.29 for all corrected VIs. This result highlights the positive impact of having consistent VIs when estimating crop leaf chlorophyll content under varying illumination conditions.

Moreover, the performance of Retinex-corrected images on leaf chlorophyll content estimation was also evaluated. As shown in Table 5, the model’s coefficient of determination ($R^2$) was 0.65 under the sunny condition and 0.61 for the variable illumination condition. The $R^2$ of models built by scaling VIs or normalized VIs decreased significantly under such weather condition. The result indicates the importance of color correction under non-uniform illumination conditions for RGB images. However, $R^2$ of the Retinex-corrected model decreased to 0.31 for the overcast condition. The result demonstrates the importance of having consistent illumination conditions during the image acquisition for crop trait estimation. When there is a change in illumination during the image acquisition, the use of illumination correction methods has the feasibility of improving the results and the variation caused due to such a non-uniform illumination condition can be addressed.

**Table 5:** Coefficient of determination ($R^2$) of the multivariable linear model of soybeans between the top-five most relevant VIs and SPAD under different illumination conditions

Illumination	Sunny	Overcast	Variable
Scaling	0.68	0.33	0.30
Normalization	0.53	0.74	0.12
ELM	0.60	0.56	–
Retinex	0.65	0.31	0.61

## Discussion

### Influence of illumination on RGB-derived vegetation indices

Figure 4 illustrates that illumination has an inevitable influence on image color. This causes, the values of the vegetation indices to change depending on the weather conditions, affecting the accuracy of crop-traits estimation. Ideally, the VIs would provide a stable value irrespective of the illumination, but we observed differences in VI under sunny and overcast conditions. Moreover, on the partially cloudy day, providing variable illumination during the flight, exceptionally high standard deviations were observed compared to the uniform conditions. This is caused by changes in solar radiation due to cloud movements.

Looking at the correlation between the scaled and normalized VIs with the SPAD measurements, variable results were obtained for the uniform conditions and a substantial decrease on the variable-illumination condition. Similar results were observed for the coefficient of determination of the multivariate linear model and the SPAD measurements.

VIs that use a ratio between color channels are theoretically more stable under variable illumination than VIs that are calculated based on a difference among channels [1, 46]. This, however, is not something that we can conclude from the results. Figure 5 does not show a clear difference between the ratio-based scaling VIs (E7-E16) and the difference-based scaling VIs (E1–E6). For both type of VIs, the standard deviation for the variable illumination is much higher than for the uniform conditions. Also, normalizing the RGB channels does not yield more stability in the VI values for the variable-illumination condition. This difference between theory and practice might be explained by the assumption of a pure brightness change, without spectral shifts. In reality, however, when cloud cover reduces the amount of direct sunlight, light is scattered more at shorter wavelengths than at longer wavelengths [10], causing different effects on different color channels, which propagates to the derived VIs. Similarly, the spectral sensitivity of the three color channels of an RGB camera is different, causing spectral shifts in the images when the intensity of illumination changes.

Thus, illumination conditions must be considered when analyzing crop physiology based on VIs using UAV-RGB imagery. In order to obtain accurate information on plant physiology throughout their growth stages using UAV imagery, it is important to mitigate the impact of illumination by algorithms and assess the sensitivity of VIs to illumination. One cannot rely simply on normalization or ratio-based VIs.

### The importance of ELM correction on chlorophyll-content estimation

As shown in Table 5, the result demonstrated the effectiveness of the ELM in reducing the impact of illumination on leaf chlorophyll content estimation under different illumination conditions. Although the scaled VIs showed a higher coefficient of determination for the sunny condition and the normalized VIs showed better performance on the overcast condition, the ELM-calibrated VIs improved the repeatability of the measurements in the sunny and overcast conditions combined. This shows the importance of illumination correction across different dates to allow comparisons over time.

Three issues must be addressed to improve the accuracy of ELM correction. The first issue is building calibration equations. This study applied a simplified and relative procedure for UAV-RGB imagery presented by Wang et al. [14] due to its applicability. However, a standardized and thorough ELM calibration follows a strict procedure [53], where radiance should be measured in physical units. The second issue is the type of reference panels. A standard reference panel should be a Lambertian surface, the apparent brightness remains the same regardless of the observer’s viewing angles. In this study, ethylene-vinyl acetate (EVA) gray-scale reference panels were used due to their low-cost and availability. Jeong’s study [49] has shown that EVA panels approximate a Lambertian surface and offer enough calibration precision to back UAV-RGB surveys. However, reference panels with standard Lambertian properties are required if more accurate radiometric data is needed. Based on the experience of fieldwork, when panels are placed in the field for long-term observation, dust and insects can easily pollute their surface, leading to changes in surface properties. Thus, keeping the panel surface as clean as possible is crucial for field investigation. Meanwhile, regular replacement of these panels is also necessary for UAV surveys. Finally, the drawback of the ELM is that it is sensitive to changing light during the flight, as calibration is done only at the start or end of the flight [54]. Light variation during flights results in inconsistent color or radiometry, thereby adding experimental error.

### The performance of retinex correction on chlorophyll-content estimation

As discussed above, partially cloudy days with sudden transitions from sun to cloud cover are the most challenging lighting conditions to handle. Without a proper correction method, all VIs were shown to be sensitive to this condition and ELM, as expected, could not deal with it. The resulting additional variation in the VIs caused the inability to predict the chlorophyll content of the plant leaves. With the use of the Retinex correction, however, the $R^2$ of the leaf chlorophyll-content estimation model and the correlation between corrected VIs and SPAD improved significantly. The result indicated that the use of a color-constancy method is helpful for crop-trait estimation under variable illumination. Neither the standard and normalized VIs nor the ELM were able to handle the challenges posed by a partially cloudy day. However, Retinex was able to effectively account for differences in weather conditions between sunny and cloudy periods, enabling crop monitoring even in these changeable conditions. Also for the sunny condition, the relationship with the SPAD measurements and the Retinex-corrected VIs was good. For the Overcast condition, however, the $R^2$ was relatively low. Further research is needed to make sure that the method performs consistently.

Compared to traditional approaches such as the use of solar irradiance sensors [11] or the placement of multiple reference panels on the ground, automated multi-scale Retinex correction offers the advantage of leveraging image content to estimate illumination properties, resulting in increased flexibility and reduced cost. The difference between the Retinex methods and previously developed image-based illumination correction algorithms for UAV multispectral images, such as [e.g., 9, 15], is that the Retinex method can estimate illumination based on images. Outside the agricultural domain, color-constancy methods are actively studied with many promising available methods [e.g., 55, 56]. In future work, the performance of other color-constancy methods could be evaluated to further improve the mitigation of variable illumination in agricultural applications. Recent developments in deep learning-based illumination estimation techniques, such as RetinexNet [57], have demonstrated promising results for close-range RGB images. These techniques need to be explored to extend for use in UAV platforms, enabling the accurate estimation of illumination conditions in diverse environments. Such investigations would represent an important contribution to the field of remote sensing and enable more effective image analysis and interpretation in UAV-based applications. Future research could also aim to investigate more reliable and generalized techniques for image-based illumination estimation on UAV platforms. Additionally, the impact of bidirectional reflectance distribution function (BRDF) effects on such methods should be further examined, particularly in scenarios with strong sunlight conditions. This will contribute to enhancing the applicability of illumination estimation in UAV-based remote sensing applications.

The results highlighted the importance of mitigating the influence of illumination on VI-based crop monitoring. Although it is suggested to collect data under consistent sunny lighting conditions to reduce the need for image post-processing, which may result in unexpected anomalies, there may be instances where data collection is necessary under variable lighting conditions. In such scenarios, the application of Retinex correction techniques can make the image data more suitable for crop monitoring, thereby increasing the time window for data collection and enabling more effective and efficient monitoring of crops.

## Conclusions

From the presented work, it can be concluded that RGB-derived vegetation indices are substantially affected by variable illumination conditions, making it difficult to compare plant traits over time when illumination conditions during data acquisition differ over time. Furthermore, the use of reference panels to correct the image with ELM correction improved the consistency of images gathered during a sunny and overcast day, improving the precision and repeatability of the estimation of chlorophyll content, compared to using non-corrected data. It should be noted that the ELM correction can not help mitigate the impact of changeable illumination during a flight as the calibration procedure usually happens only once before or at the end of the flight. At last, for UAV color images, automated multi-scale Retinex correction can help reduce the impact of variable illumination during a flight on a partially cloudy day, improving the performance of the multivariate linear model built by the top-five most relevant VIs to predict chlorophyll content. Overall, this study highlights the critical role of illumination correction and color consistency in utilizing UAV-RGB imagery for crop monitoring under diverse and variable illumination conditions. These findings emphasize the importance of accurate image analysis and interpretation in agricultural applications and underscore the potential of remote sensing technologies to support sustainable agriculture practices. Future work will focus more on exploring image-based illumination estimation and adjustment algorithms and making a comparison with sensor-based illumination correction methods. Furthermore, with the rapid development of UAV in the agricultural domain, there is a growing need to investigate the performance of image-based and sensor-based illumination correction methods in agricultural applications, including but not limited to drought monitoring and nitrogen content estimation.

## Data Availability

The datasets used and analyzed in the present study are accessible with consent from the corresponding author, which may be obtained upon making a reasonable request.
