# Correction: The impact of variable illumination on vegetation indices and evaluation of illumination correction methods on chlorophyll content estimation using UAV imagery

**DOI:** 10.1186/s13007-023-01037-7

**Published:** 2023-06-27

**Authors:** Yuxiang Wang, Zengling Yang, Gert Kootstra, Haris Ahmad Khan

**Affiliations:** 1 College of Engineering, China Agricultural University, Beijing, China; 2 Farm Technology Group, Wageningen University and Research, Wageningen, The Netherlands

**Correction: Plant Methods (2023) 19:51**

10.1186/s13007-023-01028-8

In the original publication of the article [1], the author’s name Gert Kootstra was incorrectly written as Kootstra Gert. The original article has been corrected.
